# Cystatin C, renal resistance index, and kidney injury molecule-1 are potential early predictors of diabetic kidney disease in children with type 1 diabetes

**DOI:** 10.3389/fped.2022.962048

**Published:** 2022-07-29

**Authors:** Ivana Trutin, Zarko Bajic, Daniel Turudic, Andrea Cvitkovic-Roic, Danko Milosevic

**Affiliations:** ^1^Department of Pediatrics, University Hospital Center Sestre Milosrdnice, Zagreb, Croatia; ^2^Research Unit “Dr. Mirko Grmek”, University Psychiatric Hospital “Sveti Ivan”, Zagreb, Croatia; ^3^Department of Pediatrics, University Hospital Centre Zagreb, Zagreb, Croatia; ^4^Helena Clinic for Pediatric Medicine, Zagreb, Croatia; ^5^Faculty of Medicine, Josip Juraj Strossmayer University of Osijek, Osijek, Croatia; ^6^School of Medicine, University of Zagreb, Zagreb, Croatia; ^7^Department of Pediatrics, General Hospital Zabok and Hospital of Croatian Veterans, Bracak, Croatia

**Keywords:** cystatin C, renal resistance index, urinary kidney injury molecule kIM-1, diabetic nephropathy, children, adolescent, biomarkers, diabetes mellitus type 1

## Abstract

**Background:**

Diabetic kidney disease (DKD) is the main cause of end-stage renal disease in patients with diabetes mellitus type I (DM-T1). Microalbuminuria and estimated glomerular filtration rate (eGFR) are standard predictors of DKD. However, these predictors have serious weaknesses. Our study aimed to analyze cystatin C, renal resistance index, and urinary kidney injury molecule-1 (KIM-1) as predictors of DKD.

**Methods:**

We conducted a cross-sectional study in 2019 on a consecutive sample of children and adolescents (10–18 years) diagnosed with DM-T1. The outcome was a risk for DKD estimated using standard predictors: age, urinary albumin, eGFR, serum creatinine, DM-T1 duration, HbA1c, blood pressure, and body mass index (BMI). We conducted the analysis using structural equation modeling.

**Results:**

We enrolled 75 children, 36 girls and 39 boys with the median interquartile range (IQR) age of 14 (11–16) years and a median (IQR) duration of DM-T1 of 6 (4–9) years. The three focal predictors (cystatin C, resistance index, and urinary KIM-1) were significantly associated with the estimated risk for DKD. Raw path coefficients for cystatin C were 3.16 [95% CI 0.78; 5.53; *p* = 0.009, false discovery rate (FDR) < 5%], for renal resistance index were –8.14 (95% CI –15.36; –0.92; *p* = 0.027; FDR < 5%), and for urinary KIM-1 were 0.47 (95% CI 0.02; 0.93; *p* = 0.040; FDR < 5%).

**Conclusion:**

Cystatin C, renal resistance index, and KIM-1 may be associated with the risk for DKD in children and adolescents diagnosed with DM-T1. We encourage further prospective cohort studies to test our results.

## Introduction

About 20% of the patients with type 1 diabetes mellitus (DM-T1), after 20–30 years, acquire diabetic kidney disease, which is the primary cause of end-stage renal disease in patients with DM-T1 (1–3). Therefore, it is important to detect children at risk of developing diabetic kidney disease (DKD) as early as possible. Persistent microalbuminuria and reduced estimated glomerular filtration rate (eGFR) are contemporary methods of early detection for DKD (4–6). A high percentage of regression to normoalbuminuria in children with a history of albuminuria raises the question of whether albuminuria is an valid early indicator of DKD (7–9). Cystatin C does not bind significantly to proteins and is freely, almost wholly, filtered in the glomeruli (>99%), completely reabsorbed, and degraded in the renal tubules. It does not return to the bloodstream and is not secreted in the tubules, making it a marker for the assessment of eGFR and, therefore, the risk for DKD (10). Kidney injury molecule-1 (KIM-1) as a marker of tubular damage is detected in urine before the glomerular injury, making it an early sign of DKD even before the onset of albuminuria (11). Early stages of DKD may show an increased renal resistance index (RI), which can be measured by Doppler ultrasound before the onset of microalbuminuria (12). The gold standard study design for the test of predictive value would be a prospective cohort study in which the outcome would be measured after a sufficient follow-up time. However, there is a problem with such an optimal study design in this particular case: Since DKD develops on average after 20 to 30 years, the study should last at least that long to observe a sufficient sample of patients who developed a targeted outcome (13, 14). Although it is possible to perform such a study (15, 16), it would mean having to wait 20 to 30 years for results that can improve the care of children with DM-T1. The objective of our study was to assess the association of cystatin C, RI, and KIM-1 with the estimated risk for DKD. So, we hypothesize that all three biomarkers are associated with the estimated risk for DKD.

## Materials and methods

### Study design and settings

We conducted a cross-sectional study at the Department for Pediatric Endocrinology, University Hospital Center Sestre Milosrdnice, Zagreb, Croatia, from January to December 2019. The study protocol was approved by the ethics committees of the University Hospital Center Sestre Milosrdnice and the University of Zagreb Medical School. The parents or guardians provided their written informed consent for their children’s participation. We protected the participants’ anonymity by replacing their names with numeric codes in data tables and by keeping the informed consent forms separately from the collected data forms. We performed the study in accordance with the Helsinki Declaration of the World Health Organization as revised in 2013 (17). We did not preregister the protocol, but it was used for the first author’s doctoral thesis, and it is available at the public repository of the University of Zagreb Medical School. All analysis and variables were planned before the data collection.

### Target population

The target population was children and adolescents between the ages of 10 and 18 years, diagnosed with DM-T1. The inclusion criteria were pubertal development ≥ II according to the Tanner stages, age of thelarche ≥ II degree for girls, testicular size ≥ 4 ml according to Prader staging for boys, and duration of DM-T1 ≥ 3 years if diagnosed before puberty or ≥ 2 years if diagnosed during puberty. The criterion of the stage of puberty is important due to the fact that the onset of puberty is an independent risk factor for the development of DKD (18). The exclusion criteria were acute urinary tract infection, glucocorticoid therapy, other renal diseases, orthostatic proteinuria, thyroid disease, diabetic ketoacidosis, the parvus–tardus spectrum on Doppler ultrasound, leukemia, and malignancies.

### Sample size and type

We selected a consecutive sample of participants according to the order of their arrival for a regular check-up. We calculated the required sample size before data collection for the multivariable linear regression analysis of one dependent variable to three measured predictors. We determined the minimum clinically relevant coefficient of determination for three focal predictors (FP) at R^2^ = 0.15. We set the targeted statistical power at 80% and the level of statistical significance at 0.05. Under these preconditions, 66 participants were required in the final sample. Expecting up to 10% errors in data collection, we estimated the initially required sample size to be 74 participants. We calculated the required sample size using PASS 15 Power Analysis and Sample Size Software (2017; NCSS, LLC, Kaysville, UT, United States, ncss.com/software/pass).

### Focal predictors

**FIGURE 1:**
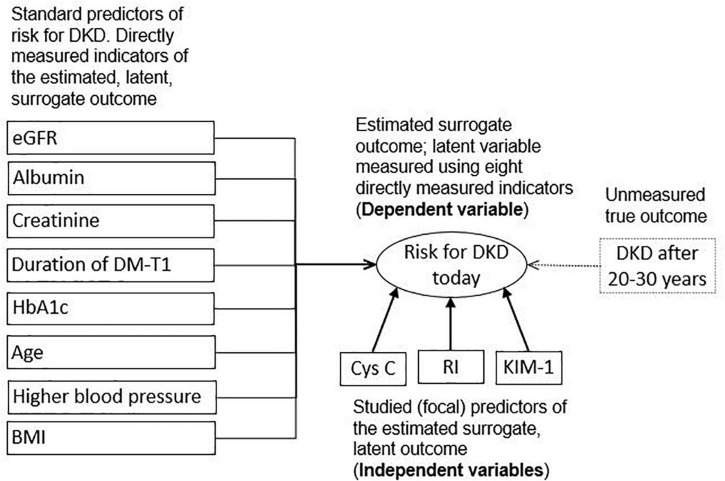
Conceptual framework; variables in solid rectangles represent directly measured variables, in oval represent a latent, indirectly measured variable, and in a dotted rectangle represent unmeasured truly targeted distal outcome; Cys C, cystatin C; RI, renal resistance index; KIM-1, kidney injury molecule-1; eGFR, estimated glomerular filtration rate; Albumin, urinary albumin; Creatinine, serum creatinine; DM-T1, diabetes mellitus type 1; HbA1c, glycated hemoglobin A1c; BP, higher blood pressure; BMI, body mass index centile.

As FP, we used three early predictive markers of DKD: cystatin C, RI, and KIM-1 (Figure 1). Cystatin C was determined using a particle-enhanced turbidimetric immunoassay (PETIA) on an Architect c8000 analyzer (Abbott Laboratories, Abbott Park, IL, United States) using the original reagent from the same manufacturer on a 4 ml-tube of anticoagulant-free blood. Only one blood sample was taken. Doppler RI was measured on a Ultrasound Philips Affiniti 50 s/n: US417D0310 (https://www.usa.philips.com/healthcare/product/HC795208/affiniti-50-ultrasound-system) with a convex C6-2 probe (2 to 6 MHz) in the back or in a side position. RI was calculated as maximum systolic velocity—minimum diastolic velocity/maximum systolic velocity. KIM-1 in a portion of the collected urine was measured by enzyme-linked immunosorbent assay (ELISA) using the Human Kidney Injury Molecule 1 (Kim-1) ELISA Kit Catalog Number. CSB-E08807h. For the quantitative determination of human kidney injury molecule 1 (Kim-1) concentrations in serum, urine, tissue homogenates.

### Outcome

The outcome was a risk for DKD estimated using eight custom predictive parameters, already documented in the literature (Figure 1). These standard indicators of elevated risk for DKD were: eGFR (Schwartz formula; ml/min/1.73 m^2^), urinary albumin (mg/g), serum creatinine (μmol/L), duration of DM-T1 (years), HbA1c (%), age (years), higher blood pressure percentile (systolic or diastolic, whichever were in higher percentile levels for the child of a given age and sex), and body mass index (BMI) (6, 19).

First morning urine samples were collected from children and adolescents diagnosed with DM-T1 for three consecutive days. Albumin concentration in the urine was determined using the immunoturbidimetric method on an Architect c8000 biochemical automated analyzer (Abbott Laboratories, Abbott Park, IL, United States) with original reagents. For each participant, systolic and diastolic blood pressure values were measured using a calibrated sphygmomanometer on three separate occasions with an appropriate cuff. Pressure gradation was determined according to the European Society of Hypertension recommendations depending on age, sex, and body height (20). HbA1c was determined on a Vantage DCA analyzer, Siemens, by the agglutination reaction method of monoclonal antibodies. The sample for the determination of HbA1c was capillary blood, 1 μL of whole blood. An alkaline picrate kinetic method determined the level of serum enzyme creatinine on an Architect c8000 analyzer with original reagents (Abbott, Abbott Park, IL, United States). One blood and three urine samples were processed in the laboratory of the University Hospital Center Sestre Milosrdnice, Zagreb, Croatia and the University Hospital Centre Zagreb, Croatia.

### Statistical analysis

We conducted the main analysis using the Multiple Indicators Multiple Causes (MIMIC) Model of structural equation modeling, which is the approach that combines confirmatory factor analysis with path analysis. The confirmatory factor analysis deals with the latent variables that are not directly measured. Although the risk for DKD can be directly measured, this can be accomplished only after a relatively long time. For this reason, we used the confirmatory factor analysis to compute the surrogate outcome measured by eight standard predictors (Figure 1). Path analysis has historically been used to model the causal relationships between directly observable variables, measured without error. However, we used it to test the hypothesis of correlation, not causation, between three FPs and one latent variable (an estimated risk for DKD indicated by eight standard predictors).

Before the data collection process, we checked the measurement and the structural part of the model’s theoretical identification (the possibility to find a unique estimate for every unknown model parameter) following a two-step identification rule. We calculated the number of observations as the number of observed variances and notredundant covariances. As a single latent factor model with eight indicators, our measurement model was theoretically identified. To lower the risk of the empirical underidentification, we used age instead of puberty stage. We skipped the puberty stage because we expected that the correlation between the two variables would be very high. We used age instead of puberty stage because age measurement was more reliable. However, we were aware that the correlation between these two variables could not be perfect. Therefore, as a kind of sensitivity analysis, we planned to repeat the model with puberty stage instead of age as the indicator of the latent criterion. For the same reason, we planned to use not the percentiles of both systolic and diastolic blood pressures but the one with a higher blood pressure percentile for the child of a given age and sex.

We conducted all these decisions and model specifications before the data collection. After we collected the data, we checked the linearity of correlations of all variables, tested their multivariate normality, and determined the existence of outliers. For the measurement part of the model, we tested the suitability of the covariance matrix for the latent factor analysis using Bartlett’s test of sphericity and the Kaiser–Meyer–Olkin test of sampling adequacy. Then, we tested the fit of the measurement part of the model to the empirical data using confirmatory factor analysis of a one-factor model measured by eight standard predictors of DKD (latent variable).

Before including eGFR, we transformed it to its inverse so that the direction of all indicators would be the same, that is, toward the higher risk for DKD. To assign the scale to the latent variable, we kept all factor loadings as free and fixed the latent factor variance to one. We estimated the model using maximum likelihood and tested its fit to the observed data using the following criteria: a statistically non-significant result of the Likelihood ratio test of the discrepancy between the original covariance matrix and the matrix estimated by the model, root mean square error of approximation (RMSEA) ≤ 0.05 with a lower bound of 90% confidence interval (CI) < 0.05 and an upper bound of 90% CI < 1.00; comparative fit index (CFI) > 0.90, Tucker-Lewis index (TLI) > 0.90, and standardized root mean squared residual (SRMR) < 0.08. In the second step, we added the recursive structural part of the model by including three FPs as potential predictors of DKD, checked the empirical identification, and tested the fit of the model using the same indicators as we used in testing the measurement part.

Only after we were convinced that the model was both theoretically and empirically identified and that it has an acceptable fit to the empirical data, we interpreted the coefficients. We calculated the standard errors and CIs/statistical significances only of the unstandardized, raw path coefficients and presented the standardized ones to aid the interpretation and comparison of the relative importance of particular predictors. As we had a relatively small sample size and some of our variables were only approximately normally distributed while we used the maximum likelihood estimation, we did a sensitivity analysis, repeating the model using robust standard errors. There were no missing data on any variable. We corrected the statistical significance for multiple testing using the Benjamini-Hochberg procedure with a false discovery rate (FDR) set at < 5%. We set a two-tailed significance level at a p-value < 0.05 and calculated all CIs at a 95% confidence level. We performed statistical data analysis using StataCorp. 2019 (*Stata Statistical Software: Release 16*, StataCorp LLC, College Station, TX, United States).

## Results

The final sample size was 75, including 36 girls and 39 boys, with a median interquartile range (IQR) age of 14 (11–16) years and a total age range from 10 to 17 years (Table 1). At the time of enrollment, the duration of DM-T1 was from 2 to 15 years, with the onset before puberty in 56 (74.7%) participants. Bartlett’s test of sphericity convincingly showed that the correlation matrix of eight standard predictors of DKD were significantly different from the identity matrix and, therefore, usable for the factor analysis (Bartlett’s test, X ^2^(28) = 109.1; *p* < 0.001). The Kaiser–Meyer–Olkin test of sampling adequacy proved that the data were suitable for factor analysis (overall 0.77; indicators ranging from 0.66 for the duration of DM-T1 to up to 0.83 for eGFR). Horn’s parallel analysis indicated the existence of only one latent factor. The measurement part of the model was theoretically and empirically identified. There were 36 observed and 16 unknown free parameters, resulting in an overidentified model with 20 degrees of freedom. Fit indexes indicated a good fit of the measurement part of the model (Likelihood ratio test of model vs. saturated model; X ^2^(20) = 21.0; *p* ≤ 0.399); RMSEA = 0.026 (90% CI 0.000; 0.105; pclose = 0.610); CFI = 0.99; TLI = 0.98; SRMR = 0.070) as well as of the final model that included the structural part with three FPs (Likelihood ratio test of model vs. saturated model; X ^2^(41) = 45.1; *p* = 0.304); RMSEA = 0.037 (90% CI 0.000; 0.090; pclose = 0.604); CFI = 0.95; TLI = 0.93; SRMR = 0.078). The final model converged in 30 iterations.

**TABLE 1:** Characteristics of the participants (*n* = 75).

	Whole sample	
Age (years), median (IQR)	14	(11–16)
**Gender, n (%)**		
boys	39	(52.0)
girls	36	(48.0)
Body mass index centile, median (IQR)	62	(41–85)
Tanner’s stages of puberty, n (%)		
II	21	(28.0)
III	12	(16.0)
IV	13	(17.3)
V	29	(38.7)
**Clinical characteristics**		
Duration of DM-T1 (years), median (IQR)	6	(4–9)
HbA1c (%), median (IQR)	7.7	(6.82–8.40)
HbA1c categorized, n (%)		
<6.5	8	(10.7)
6.5–6.9	11	(14.7)
7.0–7.4	15	(20.0)
7.5–8.4	26	(34.7)
≥8.5	15	(20.0)
**Type of insulin, *n* (%)**		
Human	5	(6.7)
Analogue	60	(80.0)
Human + analogue	10	(13.3)
Insulin dose (IU/kg), median (IQR)	0.76	(0.60–0.89)
Blood pressure (centiles), median (IQR)		
Systolic	42	(18–69)
Diastolic	55	(37–77)
**Indicators of renal function, mean (SD)**		
eGFR (ml/min/1.73 m^2^)	110	(19.2)
Urinary albumin (mg/g)	7.5	(8.40)
Serum creatinine (μmol/L)	56.2	(13.1)
**Focal predictors, mean (SD)**		
Cystatin C (mg/ml)	0.91	(0.128)
Renal resistance index	0.62	(0.040)
Kidney injury molecule-1 (ng/ml)	0.48	(0.637)

IQR, interquartile range, DM-T1, diabetes mellitus type 1; HbA1c, glycated hemoglobin A1c; IU, international units; SD, standard deviation; eGFR, estimated glomerular filtration rate.

**FIGURE 2:**
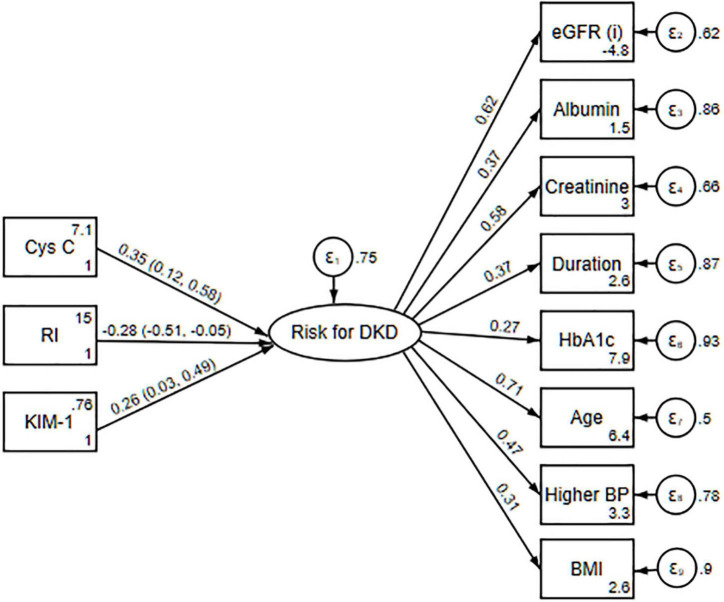
The MIMIC model with standardized estimates predicted using the maximum likelihood; latent factor variance fixed to 1; and numbers in parentheses are 95% confidence intervals. Cys C, cystatin C; RI, renal resistance index; KIM-1, kidney injury molecule-1; eGFR, estimated glomerular filtration rate; Albumin, urinary albumin; Creatinine, serum creatinine; Duration, duration of diabetes mellitus type 1; HbA1c, glycated hemoglobin A1c; BP, higher blood pressure; BMI, body mass index percentile (*n* = 75).

The three new FPs were significantly associated with the latent variable indicating the risk for DKD (Table 2 and Figure 2). Raw path (regression) coefficients for cystatin C were 3.16 (95% CI 0.78; 5.53; *p* = 0.009; FDR < 5%), for RI were –8.14 (95% CI –15.36; –0.92; *p* = 0.027; FDR < 5%), and for KIM-1 were 0.47 (95% CI 0.02; 0.93; *p* = 0.040; FDR < 5%) (Table 2). For a unit increase in cystatin C, the value of the latent variable indicating the risk for DKD increased by 0.35 (95% CI 0.12; 0.58) standard deviations. For a unit increase in RI, the value of latent variable was decreased by –0.28 (95% CI –0.51; –0.05) standard deviations, and for a unit increase in KIM-1, the value of the latent variable increased by 0.26 (95% CI 0.03; 0.49) standard deviations. The CIs and statistical significances calculated using a robust standard errors were (95% CI 0.41; 5.90; *p* = 0.024) for cystatin C; (95% CI –15.70; –0.59; *p* = 0.035) for RI, and (95% CI 0.05; 0.90; *p* = 0.030) for KIM-1. When we used puberty stage instead of age as the indicator of the latent criterion, all three observed FPs remained significantly associated with the latent variable: 2.91 (95% CI 0.60; 5.22; p = 0.014; FDR < 5%) for cystatin C, –7.41 (95% CI –14.48; –0.34; *p* = 0.040; FDR < 5%) for RI, and 0.47 (95% CI 0.03; 0.91; *p* = 0.036; FDR < 5%) for KIM-1.

**TABLE 2:** The final MIMIC model coefficients (*n* = 75).

	Raw coefficients	(95% CI)	*p*	Standardized coefficients
**Structural part**				
Cystatin C	3.16	(0.78; 5.53)	0.009*	0.35
Renal resistance index	–8.14	(–15.36; –0.92)	0.027*	–0.28
KIM-1	0.47	(0.02; 0.93)	0.040*	0.26
**Measurement part**				
eGFR (inverse)	10.22	(6.25; 14.18)	<0.001	0.62
Urinary albumin	2.67	(0.78; 4.55)	0.006	0.37
Serum creatinine	3.25	(1.86; 4.63)	<0.001	0.58
Duration of DM-T1	1.11	(0.34; 1.88)	0.005	0.37
HbA1c (%)	0.24	(0.01; 0.47)	0.042	0.27
Age (years)	1.60	(1.03; 2.17)	<0.001	0.71
Higher blood pressure	9.46	(4.42; 14.51)	<0.001	0.47
Body mass index (centile)	7.46	(1.27; 13.66)	0.018	0.31

Confidence intervals and statistical significances were calculated only on the row of unstandardized coefficients.

KIM-1, kidney injury molecule-1; eGFR, estimated glomerular filtration rate; DM-T1; diabetes mellitus type 1; HbA1c, glycated hemoglobin A1c; CI, confidence interval; *p*, statistical significance of the coefficients.

*False discovery rate < 5%.

## Discussion

Our study indicated that cystatin C, RI, and KIM-1 might be associated with the risk for DKD in children and adolescents diagnosed with DM-T1, at least to the extent that it is validly indicated by the eight standard predictors: eGFR, urinary albumin, serum creatinine, duration of DM-T1, HbA1c, age, higher blood pressure percentile (systolic or diastolic, whichever were in higher percentile levels for the child of a given age and sex), and BMI.

These three glomerular, vascular, and tubular markers are rarely simultaneously studied and are not part of the guidelines for screening for DKD in children with DM-T1 (5). The significant association between cystatin C and the latent variable indicating the risk for DKD is yet another confirmation of the probable value of cystatin C in the prediction of DKD, which has been documented (21–25). Cystatin C has been shown to be a significant marker of renal impairment prior to microalbuminuria (26–30).

Most studies did not find an association between KIM-1 in urine and GFR, explaining this by associating GFR with glomerular damage and emphasizing proximal tubular damage as a separate stage of DM-T1 development (31, 32). Association of KIM-1 in the sample of normoalbuminuric children and adolescents with DM-T1, with the latent variable indicating the risk for DKD, may also suggest early tubular damage, even before the onset of glomerular damage and microalbuminuria (31). This evidence supports the opinion that the KIM-1 levels in urine are not elevated in patients with DM-T1 because of “toxicity of albuminuria due to glomerular damage” but are elevated because of damage to the proximal tubules *per se*. Similar findings were reported before (6, 33). Elevated urinary KIM-1 levels in microalbuminuric patients with DM-T1 show a decrease in urinary KIM-1 values, concomitant with regression of microalbuminuria in normoalbuminuria (34).

A statistically significant inverse association of RI and a latent variable indicating the risk for DKD in our study does not seem logical. It contradicts the findings of several other studies (8, 35). Some researchers also did not find a statistically significant difference in RI values in children with DM-T1 and in the healthy population and no association between albuminuria and RI. Therefore, the authors concluded that RI is not a good predictor of early renal impairment in patients with DM-T (36). Our result may be interpreted as the effects of some unmeasured confounding factors or that RI inverse association may be the result of early hyperfiltration and consequent renal preglomerular vasodilation and lower RI compensatory mechanism/s at a very early renal damage (19, 36–40). Microalbuminuria is considered to be the earliest marker of DKD development and is often associated with significant glomerular damage and thickening of the glomerular basement membrane, along with hyperfiltration (41). We strongly encourage repeating the analysis of RI in children with DM-T1. Using a combination of indicators of glomerular (serum cystatin C), vascular (Doppler RI), and tubular (KIM-1 in urine) impairments, it probably may be possible to improve a model for early detection of renal impairment in children with DM-T1. Normoalbuminuric children with DM-T1 with normal renal function may have symptoms suggestive of early renal impairment, and determination of the combination of serum cystatin C, KIM-1 in urine, and Doppler RI may be important in the future for preventive and therapeutic actions in patients. Renal biopsy results of patients who have not yet developed microalbuminuria show structural changes that include glomerulus and tubulointerstitium: thickening of the glomerular and tubular basement membranes, diffuse expansion of the mesangia, and hyalinosis of the afferent and efferent arterioles (42). It was also found in normoalbuminuric children with DM-T1 in prepuberty, with an average disease duration of five to eight years (42).

We propose several directions for future studies. First, to test the reproducibility of our results with a similar study design on similar populations of children with DM-T1. This should be done to test the credibility of our findings and to estimate the heterogeneity and generalizability of the effects we observed. Second, suppose the results of these studies confirm our findings to an acceptable extent, we propose a cross-sectional study on the samples large enough to enroll a sufficient number of participants with microalbuminuria to test the current standard. Then, we propose a case-control study with microalbuminuria as the primary outcome. Finally, we propose a prospective cohort study that will directly measure the incidence of DKD after sufficient follow-up time and using a proper time-to-event/survival analysis. Since we used relatively complex statistics in a single statistical package, we invite other researchers to download our data and check the replicability in Stata and other available software solutions like LISREL, Mplus, SAS module CALIS, Lavaan in R, and so on.

Of course, we hope that our results will survive such verifications; however, we will be happy if colleagues refute and correct our mistakes. Future studies should investigate other glomerular, vascular, and tubular markers as predictors of DKD as well.

### Limitations of the study

The main limitation of this study is its cross-sectional design instead of a prospective cohort one with the longitudinal follow-up of patients. The consequent limitation is the usage of the surrogate, latent variables as the non-direct measured outcomes instead of the confirmed DKD as the truly targeted outcome (Figure 1). In other words, based on this study design, it is not possible to validly determine whether the variations in eight standard predictors of risk for DKD and in the three FPs are truly due to the chronic changes with the predictive validity for the future DKD or if they just represent the transient states with no long-term relevant health consequences. For example, we could not reliably and validly rule out the possibility that KIM-1 was acutely or transiently elevated due to any acute kidney injury or other effects on the proximal tube, such as the usage of non-steroid anti-inflammatory drugs or other medications, hemodynamic effects, and dehydration. Given that the included patients underwent regular control treatment in the hospital, hemodynamic effects/dehydration were minimized, but they were not reliably excluded. We cannot speculate on the direction and the magnitude of the bias caused by this, and the only valid solution to the dilemma is in future prospective studies that will directly measure the target outcome.

The second limitation is using the maximum likelihood estimation. We tested the model’s fit to the matrices of the original covariances. However, the average value of our surrogate outcome, the latent factor measured by standard DKD indicators, is likely to have lower predictive validity for the actual future DKD. The elevated rather than the average value should be modeled, for example, the 75th percentile of the surrogate latent outcome. Unfortunately, we are not aware of the structural equation modeling method that would construct the outcome based on manifest indicators and still, in the structural part of the same model, would do something like quantile regression to some elevated value of that criterion. This statistical analysis, or our knowledge, limitation, lowered the predictive validity of our analysis. We tried to model the latent criterion by logistic regressions of the indicators binarized to their values below and above the 75th percentile (indicating the elevated risk for DKD), but these models did not converge, probably because of the insufficient sample size.

The third limitation is that the structural part of our model assumes that cystatin C, RI, and KIM-1 were measured without error. Balancing ethical arguments (discomfort and other, albeit small, risks) and the expected benefit for the reliability of cystatin C measurement, we concluded that it is optimal to do only one measurement. However, it is known that cystatin C have relatively wide physiological fluctuations within the normal range and between measurements, and therefore, using only one blood sample as we did may lower the reliability of the measurement.

Although we have no empirical and rational reason to believe that the association of the three new FP with the latent factor measured by different standard DKD predictors differs between different countries, regions, and children or adolescents treated in different types of institutions, such a possibility should not be overlooked. We could not assume with the least reliable certainty whether the unicentric nature of our study design worked in favor of or against the null hypothesis or how large the weakness of generalizability that may have caused it could be. The only solution is to replicate the study in a multicenter setting.

## Conclusion

Cystatin C, renal resistance index, and KIM-1 might be associated with the risk for DKD in children and adolescents diagnosed with DM-T1, as indicated by eight standard predictors. A future prospective cohort study should include these three indicators of the elevated risk for DKD and test their predictive validity.

## Data availability statement

The raw data supporting the conclusions of this article will be made available by the authors, without undue reservation.

## Ethics statement

This study involving human participants was reviewed and approved by Ethics Committee of University Hospital Centre “Sisters of Charity”, Zagreb, Croatia. Written informed consent to participate in this study was provided by the participants’ legal guardian/next of kin.

## Author contributions

IT, DT, ZB, and DM conceived and designed the study. IT collected the data and was responsible for the submission of the manuscript. IT, ZB, and DM developed the plan for statistical analysis. IT, DT, and ZB drafted the manuscript. All authors provided the study design, data interpretation, and provided critical revisions of the manuscript.
